# Odor-Binding Protein 2 in *Apis mellifera ligustica* Plays Important Roles in the Response to Floral Volatiles Stimuli from Melon and Tomato Flowers

**DOI:** 10.3390/ijms26073176

**Published:** 2025-03-29

**Authors:** Jiangchao Zhang, Weihua Ma, Yue Zhang, Surong Lu, Chaoying Zhang, Huiting Zhao, Yusuo Jiang

**Affiliations:** 1 College of Animal Science, Shanxi Agricultural University, Jinzhong 030600, China; zjczjczjc1997@163.com (J.Z.); 18883628786@163.com (Y.Z.); lusurong2103@163.com (S.L.); wsqzcy0@163.com (C.Z.); 2 College of Horticulture, Shanxi Agricultural University, Taiyuan 030000, China; 3 College of Life Sciences, Shanxi Agricultural University, Jinzhong 030600, China; zhaohting@126.com

**Keywords:** *Apis mellifera ligustica*, expression profiles, floral volatiles, ligand binding, odor-binding protein, RNAi

## Abstract

Honeybee olfaction can influence foraging behavior and affect crop pollination. Odor-binding proteins play a vital role in honeybee olfactory perception. A previous study based on the antennal transcriptome of *Apis mellifera ligustica* in melon and tomato greenhouses revealed that *AmelOBP2* is highly expressed. Therefore, we aimed to further investigate the olfactory recognition mechanism of honeybees by detecting the expression levels and binding ability of AmelOBP2 to floral volatiles of melon and tomato flowers. The results show that *AmelOBP2* mRNA was highly expressed in the antennae of honeybees, and its protein expression was highest in the antennae at 20 days of age and was higher in the melon greenhouse. The binding ability of AmelOBP2 to floral volatiles of melon was stronger than that of tomato. AmelOBP2 had a stronger binding ability with aldehydes in melon floral volatiles and with terpenes and benzenes in tomato floral volatiles. After feeding with siRNA, the electroantennogram response of honeybees to E-2-hexenal, E-2-octenal, and 1-nonanal decreased markedly, confirming the role of AmelOBP2 in the recognition of melon and tomato floral volatiles. These results elucidate the molecular mechanisms underlying honeybee flower-visiting behavior and provide a theoretical reference for regulating the behavior of honeybees using plant volatiles.

## 1. Introduction

Floral volatiles as olfactory signals play a key role in mediating interactions between plants and pollinating insects and affect plant reproduction [1,2,3]. Floral volatiles have a significant impact on the foraging behavior of pollinating insects by providing information, such as plant location and the availability and quality of food [4,5,6]. Most nocturnal bees prefer flowers with strong fragrances composed of aromatic compounds such as eugenol, methyl salicylate, and 1-Octanol [7]. While honeybees respond to key volatiles such as nonanal and α-phellandrene in crops like kiwi [8], their pollination efficiency varies substantially across plant species. However, honeybees exhibit low pollination efficiency and unstable floral-visiting behavior in tomato cultivation compared to melons [9,10,11,12].

This behavioral discrepancy may stem from differential responses to species-specific floral volatile profiles. Our preliminary investigations revealed distinct electrophysiological (EAG) and behavioral responses in honeybees exposed to tomato and melon floral volatiles [13,14]. Additionally, the transcriptome sequencing of honeybee antennae at different developmental stages in melon and tomato greenhouses identified *AmelOBP2* as an odorant-binding protein (OBP) gene with high expression. OBP2 could bind to some plant volatiles with strong affinity [15].

The insect olfactory system employs specialized proteins, includingOBPs, chemosensory proteins (CSPs), and odorant receptors (ORs) to decode chemical signals [16,17]. Among these proteins, OBPs play an important role in recognizing olfactory stimuli from odorants or volatiles [18,19] and are engaged in other developmental and physiological functions [20]. Different OBPs selectively bind odorants and/or pheromones. As a major pollinating insect, studies on the function of OBPs in honeybees have reported that OBPs such as Amel/AcerOBP1 show strong binding with major queen pheromones [21,22]. Amel/AcerOBP2 is apt to bind with floral volatiles and nonsexual pheromones [23,24]. AmelOBP21 can bind with the queen mandibular pheromone [25]. AcerOBP6 has binding affinity with queen pheromone, worker pheromone, and floral volatiles [26].

In order to determine whether floral volatiles are responsible for the unstable floral-visiting behavior of bees towards tomatoes and to investigate the potential mechanism of the response of OBP2 to the volatiles of melon and tomato flowers, we conducted this study. The study assessed mRNA and protein expression levels of AmelOBP2, analyzed their binding affinity to floral volatiles using fluorescence binding assays, and evaluated the impact of AmelOBP2 knockdown via siRNA on honeybee electroantennogram (EAG) responses. This study provides a theoretical reference for regulating the behavior of bees using plant volatiles, contributing to our understanding of honeybee foraging behavior and plant–pollinator interactions.

## 2. Results

### 2.1. Expression Pattern Analysis of AmelOBP2

**Figure 1:**
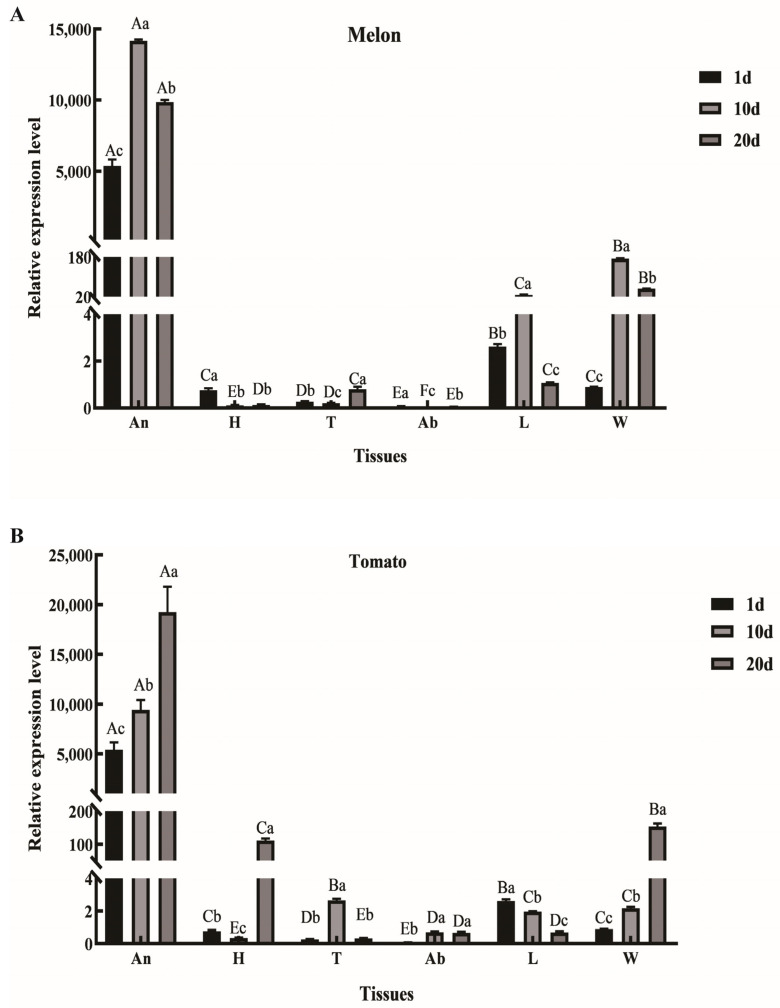
The mRNA expression of *AmelOBP2* of *Apis mellifera ligustica*. The honeybees in the melon (**A**) and tomato (**B**) greenhouse for 1, 10, and 20 days, and the relative gene expression of *AmelOBP2* in various tissues. An, antenna; H, head; T, thorax; Ab, abdomen; L, leg; W, wing; 1–20 d, number of days old for *A. m. ligustica*. Different uppercase letters indicate significant differences in expression among the different tissues at the same stage (*p* < 0.05), and different lowercase letters indicate significant differences in expression among the different stages in the same tissue (*p* < 0.05) (Duncan’s method).

**Figure 2:**
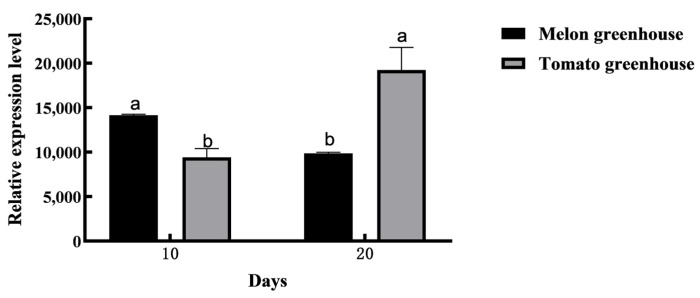
The mRNA expression of *AmelOBP2* in antennae at different developmental stages of *A. m. ligustica* in melon and tomato greenhouses. Different lowercase letters indicate significant differences (*p* < 0.05) (*t*-test).

We analyzed the expression of AmelOBP2 in honeybees from melon and tomato greenhouses using qRT-PCR and Western blotting. At the mRNA level, the results showed that *AmelOBP2* was expressed in all tissues of honeybees of different ages, with significantly higher expression levels in the antenna tissues than in other tissues (*p* < 0.05) (Figure 1). The expression of *AmelOBP2* in the antennae of honeybees in the melon greenhouse was significantly higher than that of honeybees in the tomato greenhouse at 10 d of age (*p* < 0.05), whereas there was a reversal of this result at 20 d of age (Figure 2).

**Figure 3:**
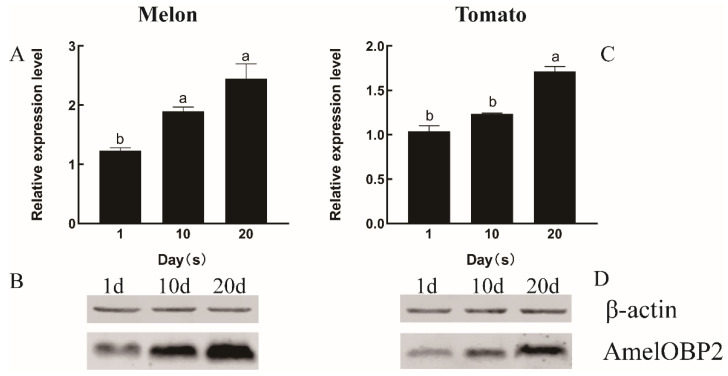
The protein level of AmelOBP2 in the antennae at different developmental stages of *A. m. ligustica* in melon and tomato greenhouses. (**A**,**B**) Melon greenhouse; (**C**,**D**) tomato greenhouse. Different lowercase letters indicate significant differences (*p* < 0.05) (*t*-test).

The protein level of AmelOBP2 in honeybee antennae showed a similar trend of increasing with age in both the melon and tomato greenhouses, with the highest expression level observed at 20 d of age. In the melon greenhouse, the protein level of AmelOBP2 in the antennae of 10-day-old and 20-day-old honeybees was significantly higher than that in 1-day-old honeybees (*p* < 0.05). Similarly, in the tomato greenhouse, the protein level of AmelOBP2 in the antennae of 20-day-old honeybees was significantly higher than that in 1-day-old and 10-day-old honeybees (*p* < 0.05). Moreover, we also found that the protein level of AmelOBP2 in the melon greenhouse was consistently higher than that in the tomato greenhouse across all the time points tested (Figure 3).

### 2.2. AmelOBP2 Binding Properties Analysis

#### 2.2.1. Recombinant AmelOBP2

**Figure 4:**
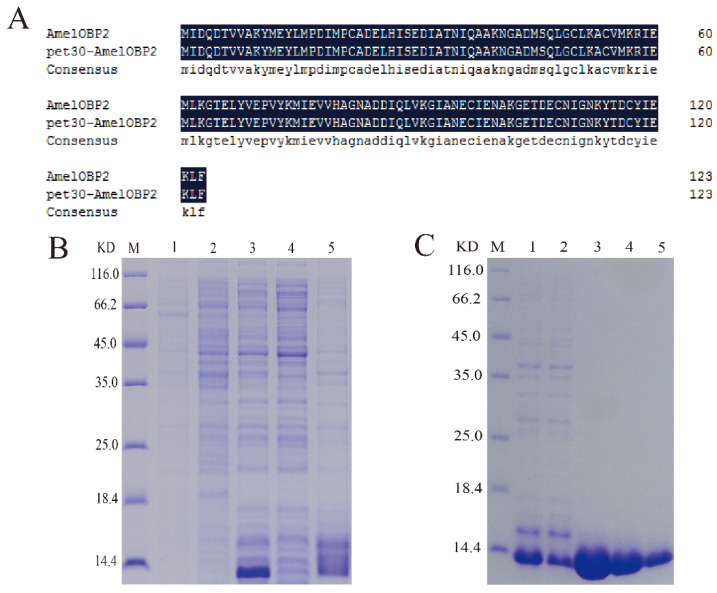
Preparation and purification of the recombinant protein AmelOBP2. (**A**) Alignment of the recombinant expression plasmid sequence with the target protein sequence; (**B**) SDS-PAGE analysis for protein expression identification; (**C**) SDS-PAGE analysis for AmelOBP2 protein purification. M: protein molecular weight standard; B1: pET-30a induction; B2: no induction; B3: after induction; B4: supernatant after induced fragmentation; B5: precipitate after induced fragmentation. C1: post-crushing treatment sample; C2: effluent; C3–5: eluent.

The pET-30a (+)/AmelOBP2 vector was successfully constructed; AmelOBP2 was successfully expressed in the form of an inclusion body after induction by the *E. coli* prokaryotic expression system, and the target protein was obtained after purification (Figure 4).

#### 2.2.2. AmelOBP2 Binding Curve with 1-NPN

**Figure 5:**
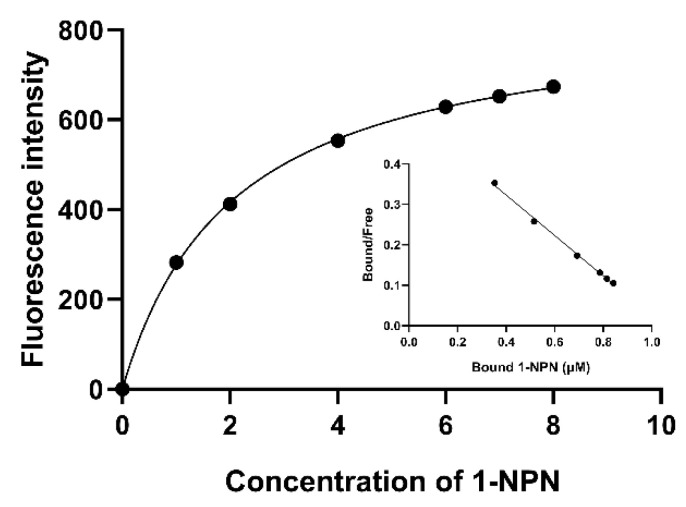
Binding curve and Scatchard plot (insert) of 1-NPN to AmelOBP2.

At an excitation wavelength of 337 nm, no clear fluorescence signal was observed from the AmelOBP2 protein working solution. By adding 3 μL of the 1-NPN solution to the protein working solution successively, the maximum fluorescence intensity was found at 405 nm, and the fluorescence intensity increased with the increase in the probe concentration. The resulting data were then fitted using regression and Scatchard processing to produce a combination curve and linear equation for AmelOBP2 and 1-NPN (Figure 5). The linear equation was y = −0.5000x + 0.5231; the correlation coefficient (R^2^) was 0.997; and the binding constant between AmelOBP2 and the probe was 2 μM, indicating that we could proceed to the next step.

#### 2.2.3. Ability of AmelOBP2 to Bind to Melon and Tomato Floral Volatiles

**Figure 6:**
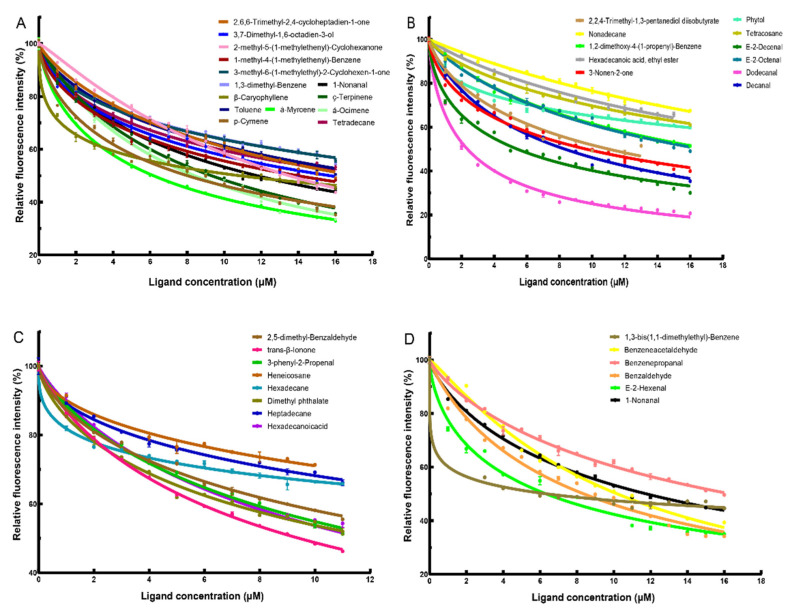
Competitive binding curves of AmelOBP2 to floral volatiles of melon and tomato. (**A**) Competitive binding curves of AmelOBP2 to floral volatiles of tomato; (**B,C,D**) Competitive binding curves of AmelOBP2 to floral volatiles of melon.

The binding ability of the recombinant protein AmelOBP2 to the 14 tomato and 25 melon floral volatiles was determined using a fluorescence competitive binding assay. The IC50 values of the odor standard compounds obtained from the fluorescence competitive binding curve (Figure 6) and the binding constant Ki between AmelOBP2 and the odor standard compounds are shown in Table 1.

We determined that AmelOBP2 was bound to all tested standard floral volatile odor compounds. Among the melon floral volatiles, AmelOBP2 had the strongest binding ability to aldehydes such as dodecanal (K_i_ = 1.05 µM). However, it exhibited a relatively weak binding ability toward alkanes such as heneicosane and hexadecane. Among the tomato floral volatiles, AmelOBP2 showed the strongest binding ability to terpenes such as alpha-myrcene, β-ocimene, gamma-terpinene, β-caryophyllene, and p-cymene (Ki = 0−5 µM).

### 2.3. Effect of siRNA on AmelOBP2 mRNA Expression

**Figure 7:**
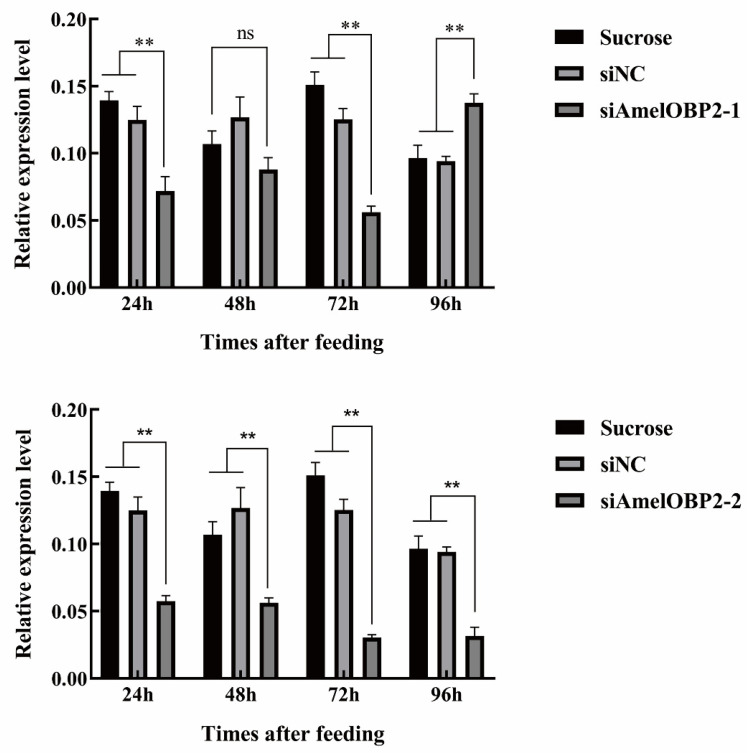
Effect of feeding honeybees siRNA on *AmelOBP2* mRNA expression level. ** indicates highly significant difference (*p* > 0.01); ns indicates that the difference is not significant (*p* < 0.05).

Within 24–96 h of siRNA feeding, the optimal siRNA and silencing times were determined based on the change in *AmelOBP2* mRNA expression (Figure 7). Compared with the sucrose and siNC groups, the siAmelOBP2-1 group (79 bp) showed a significant decrease in *AmelOBP2* expression at 72 h, and the silencing efficiency was 62.86% (*p* < 0.01); the siAmelOBP2-2 group (329 bp) showed a significant decrease in the mRNA expression level of AmelOBP2 (*p* < 0.01). The silencing efficiencies at 24, 48, 72, and 96 h after siRNA feeding were 58.87%, 47.35%, 79.83%, and 67.19%, respectively. Therefore, the optimal siRNA and silencing times were siAmelOBP2-2 and 72 h after feeding, respectively.

### 2.4. EAG Analysis Before and After RNAi

To clarify the physiological function of amelobp2 in the detection of floral volatiles in honeybees, we selected the following five volatiles: e-2-hexenal, e-2-octenal, 1-nonanal, 2-methyl-5-(1-methyl ethenyl)-cyclohexanone, and methyl benzene. Specifically, e-2-hexenal and e-2-octenal are volatiles of melon flowers; 2-methyl-5-(1-methyl ethenyl)-cyclohexanone and methyl benzene are volatiles of tomato flowers; and 1-nonanal is both a melon flower volatile and a tomato flower volatile. The antennal electrical activity of the honeybees was measured after feeding with siAmelOBP2. The results are summarized in Table 2. Compared with the EAG response of bees in the sucrose and siNC groups, the response of honeybees fed siAmelOBP2 was significantly decreased, and the silencing efficiencies for e-2-hexenal, e-2-octenal, and 1-nonanal were 34.64%, 37.28%, and 55.20%, respectively (*p* < 0.05). The EAG response to 2-methyl-5-(1-methyl ethenyl)-cyclohexanone and methyl benzene also decreased in honeybees fed siAmelOBP2, but this difference was not significant, and the silencing efficiencies were 32.48% and 28.76%, respectively (*p* > 0.05).

## 3. Discussion

Insect OBPs have highly conserved structures, and their expression patterns at the mRNA and protein levels reflect their physiological functions to some extent [27,28]. Previous studies suggested that OBPs are specifically expressed in the antennae, which has been verified in multiple insect species [29,30,31,32]. Furthermore, the expression of OBPs in the antennae, which is one of the main olfactory organs, may be associated with an insect’s perception of VOCs in the environment [33,34]. In this study, AmelOBP2 was highly expressed in honeybee antennae, indicating that it may play a key role in the olfactory recognition of floral volatiles. Amel/AcerOBP2 has shown strong binding with floral volatiles [15,23,24].

Honeybees are social insects, and workers change their social division of labor within the colony as they age [35]. We determined that the expression of *AmelOBP2* changed with developmental age. This is consistent with the expression patterns of most *AcerOBPs* and *AmelOBPs* [26,33]. In the melon greenhouse, we found that the expression of *AmelOBP2* at 10 d of age was significantly higher than that at 20 d. In a study of the expression pattern of *AcerASP2*, a similar phenomenon was observed [36]. This pattern may be attributable to the transition from nurses to foragers at around 10 d of age when honeybees start secreting large amounts of beeswax to build their combs [37]. We analyzed the protein level of AmelOBP2 and found that the protein expression did not completely correlate with the mRNA expression pattern. This might be because the expression of eukaryotic genes is not only regulated at the transcriptional level but is also affected by post-transcriptional regulation, translation, and post-translational regulation, such as protein processing and modification [33]. In the present study, the protein level of AmelOBP2 in the antennae of honeybees in the two greenhouses differed. In honeybees of the same age, the expression level of AmelOBP2 was higher in honeybees in the melon greenhouse than in honeybees in the tomato greenhouse. Studies have shown that honeybees do not prefer foraging on flowers from nightshade plants such as tomatoes because of their distinctive odors [12]. We hypothesized that the different AmelOBP2 expression levels in honeybees in the two greenhouses might have been caused by different floral volatiles; however, this requires further verification.

In the fluorescence competitive binding test, AmelOBP2 could widely bind to the floral volatiles of melon and tomato, indicating that AmelOBP2 plays a crucial role in the process of locating flowers using the olfactory system. This is consistent with the finding that AmelOBP2 is expressed in high abundance in the antennae at both the mRNA and protein levels.

We found that AmelOBP2 has different abilities to bind to various floral volatiles. In a study of melon floral volatiles, AmelOBP2 showed the strongest binding ability to aldehydes, such as dodecanal, e-2-hexenal, and benzaldehyde. Aldehydes are commonly present in flowering plants and are the major components of melon flower fragrance [14]. Benzaldehyde and e-2-hexenal are abundant in melon flower fragrance, whereas dodecanal is relatively less abundant [14]. During the pollination process of melon flowers, honeybees can detect trace amounts of VOCs and effectively utilize high concentrations of VOCs. Furthermore, we found larval pheromone components, such as methyl palmitate and ethyl palmitate, in the floral volatiles of melon flowers [14]. Larval pheromones can regulate the feeding behavior of nurse honeybees [33]. After treating honeybee colonies with methyl palmitate and ethyl palmitate, it was found that these two volatiles could inhibit the ovarian development of workers, promote the development of the hypopharyngeal gland, and stimulate foragers to collect pollen [38,39,40]. Notably, AmelOBP2 exhibited binding ability toward both compounds, suggesting that AmelOBP2 also plays a role in larval feeding and colony maintenance. Among tomato floral volatiles, we found that terpenes, such as β-ocimene and β-caryophyllene, showed the strongest binding ability to AmelOBP2. In particular, β-caryophyllene has the highest content, and it is widely present in floral volatiles and strongly attracts *Apis cerana cerana* [41]. AmelOBP2 likely plays an important role in tomato flower pollination [13]. A smaller Ki value indicated a stronger binding ability of AmelOBP2 to two floral volatiles. Compared to the binding ability of AmelOBP2 to the two floral volatiles, the binding constant of the protein to dodecanal in melon floral volatiles can reach 1.05 µm, and the binding ability of the protein to e-2-decenal, e-2-hexenal, 1,3-bis (1,1-dimethyl ethyl)-benzene, and benzaldehyde is stronger. In general, AmelOBP2’s binding ability to melon floral volatiles was stronger than its binding ability to tomato floral volatiles, which was consistent with the higher AmelOBP2 expression in honeybees in the melon greenhouse.

Whether odors with a strong binding ability to OBPs play a role in insect chemical detection (such as recognizing floral odors and locating hosts) requires further verification through in vivo RNAi experiments. In the RNAi test, feeding honeybees 329 bp siAmelOBP2-2 for 72 h showed the best silencing effect. We found that siRNA with longer chains exhibited better silencing effects. After feeding with siAmelOBP2, the EAG response of honeybees to e-2-hexenal, 2-methyl-5-(1-methyl ethenyl)-cyclohexanone, e-2-octenal, 1-nonanal, and methyl benzene decreased, suggesting that AmelOBP2 is involved in the recognition mechanisms of these five compounds. The EAG response to e-2-hexenal, 1-nonanal, and e-2-octenal decreased significantly, indicating that AmelOBP2 plays a key role in the recognition of these three compounds. The result provides a valuable reference for honeybees’ preference for flower visiting and future research on behavioral regulation using plant volatiles.

In this study, AmelOBP2 showed the strongest binding ability to aldehydes and terpenes. We hypothesize that the targeted application of these compounds to tomato flowers could enhance pollination efficiency by attracting honeybee visitors. However, this potential strategy requires rigorous validation through in vivo behavioral experiments with honeybee colonies to simultaneously assess both efficacy (increased visitation rates and pollination success) and biosafety (the absence of adverse ecological impacts on pollinators or plant physiology).

## 4. Materials and Methods

### 4.1. Insects

Honeybees (*Apis mellifera ligustica*) were collected from colonies in melon and tomato greenhouses in Dongshandi Village, Shanxi Province, China. Capped brood combs near eclosion were selected from the colonies and transferred to the laboratory incubator (65–70% relative humidity; 34 ± 1 °C). Newly hatched honeybees were labeled with nontoxic paint and reintroduced to the greenhouse hive for development. A total of 300 honeybees were collected at 1, 10, and 20 d of age and randomly divided into 3 groups. The antennae, head (antennae excision), thorax, abdomen, legs, and wings were separated and ground into powder with liquid nitrogen. The samples were stored at −80 °C for subsequent testing.

### 4.2. AmelOBP2 Expression Pattern Analysis

#### 4.2.1. qRT-PCR

Total RNA and cDNA were obtained according to Du *et al.* [33]. The obtained cDNA was subjected to qRT-PCR using the SYBR fluorescent dye method (Takara, Japan) (Table A1). The primers were synthesized by Sangon Biotech (Table A2). Data processing refers to Livak and Schmittgen [42] (2^−ΔΔCt^ method).

#### 4.2.2. Purification of AmelOBP2

Antenna cDNA from a one-day-old honeybee was used as the template for PCR amplification (Table A3). PCR-recovered products were cloned into pET-30a vectors (Novagen, Madison, WI, USA) by enzyme digestion and ligand reaction (Table A4). The recombinant plasmid was transformed into Trans1-T1 chemically competent cells; then, the correct plasmid was selected and transformed into *E. coli* BL21(DE3) cells to detect massive expression (TransGen Biotech Co., Ltd., Beijing, China). Single colonies were selected and inoculated into LB liquid medium (50 μg/mL Kan^+^) at 37 °C and 220 rpm for shock culture. When the absorbance OD_600_ of the bacteria was 0.6–0.8, IPTG was added to a final concentration of 0.2 mM to induce the expression of the recombinant fusion protein. The dissolution of the recombinant proteins was analyzed using SDS-PAGE. The body proteins included were purified using the Ni-IDA-Sepharose Cl-6B affinity chromatography column (Sangon Biotech, Shanghai, China). After dialysis and concentration, the proteins were freeze-dried for further use.

#### 4.2.3. Western Blot

Samples (0.1 g) were added to 1 mL of the protein extract solution (PMSF: RIPA = 1:1000; Boster, Wuhan, China) to extract the total protein. Next, the protein samples were separated using SDS-PAGE (60 V, 40 min). The protein was then transferred to 0.22 μm cellulose nitrate NC film at 100 V for 90 min (Boster, Wuhan, China). After enclosing the NC film, it was placed in the diluent solution (OBP2, 1:1000; β-Actin, 1:500) overnight in a shaker at 4 °C. The NC film was incubated in a secondary antibody IRDye 800CW Goat anti-Rabbit IgG diluent (LI-COR, Lincoln, Nebraska, USA) (1:20,000) and then exposed using an Odyssey^®^ CLx Imaging System (LI-COR, Lincoln, Nebraska, USA). Image J was used to detect the gray values of Western-blot assay data. The ratio of gray values of OBP2 to β-Actin was the relative expression level of the protein.

### 4.3. Fluorescence Competitive Binding Test

The ability of AmelOBP2 to bind to floral volatiles in tomato and melon greenhouses was determined using an RF-5301PC fluorescence spectrophotometer (Shimadzu, Japan) with 1-Naphthylthiourea phenanthroline (1-NPN) (TCI, Japan) as the fluorescence probe (Table A5). The parameters of the RF-5301PC fluorescence spectrophotometer are set as follows: the emission wavelength range is 360–500 nm, the excitation wavelength is 337 nm, and the slit width is 3 nm. This process was similar to that described by Du et al. [33]. Data were processed using GraphPad Prism 8.0. The binding constant (K_i)_ of AmelOBP2 and odor volatiles were computed using the following equation [26]:

K_i_ = [IC50]/(1 + [1-NPN]/K_1-NPN_)

IC50 refers to the concentration of the odorant at which the fluorescence intensity of the AmelOBP2/1-NPN complex was reduced by 50%. 1-NPN refers to the concentration of unbound 1-NPN, while K_1-NPN_ refers to the binding constant of the AmelOBP2/1-NPN complex. The smaller the K_i_, the stronger the binding ability of the substance to the recombinant protein. K_i_ values ranging from 0 to 5µM indicate strong binding ability with AmelOBP2.

### 4.4. Detection of RNAi Silencing Effect

Honeybees were collected at 20 d of age from an apiary at the College of Animal Science, Shanxi Agricultural University. Honeybees were randomly divided into 4 groups (n = 40) and starved in an incubator for 30 min (65–70% relative humidity; 28 ± 1 °C). Honeybees were fed with siAmelOBP2 (8 μg per bee dissolved in sucrose solution); the siAmelOBP2 are recorded as siAmelOBP2-1 (79 bp) and siAmelOBP2-2 (329 bp). We used siNC and 30% sucrose solution as the controls. After feeding, the honeybees were placed back in the incubator. At 24, 48, 72, and 96 h, the honeybees were captured randomly in each group, and the silencing effect of RNAi was assessed using qRT-PCR.

### 4.5. EAG Recording

Based on the results of the fluorescence competitive binding tests and our previous research involving EAG tests [13,14], odor-standard compounds were identified for testing (Table A5). The optimal siRNA and silencing time were determined by an experiment and fed to honeybees. The EAG response was measured using an EAG insect antennal potential measurement system (Syntech, Netherlands) [13]. Each measurement was repeated using three antennae. The results of the antennal potential measurements were expressed as the EAG relative response value (rEAG), which was calculated as follows [14]:

rEAG = (EAG (X) − EAG (std))/EAG (std)(1)

EAG (X) represents the response amplitude (mV) of the EAG to a given compound, while EAG (std) represents the response amplitude (mV) of the EAG to the reference liquid paraffin at each recording stage.

### 4.6. Statistical Analysis

One-way ANOVA and Duncan’s method were used to assess statistical significance (SPSS 21.0), with *p* < 0.05 considered statistically significant. The final results were expressed as the mean ± standard error.5. Conclusions

In summary, AmelOBP2 is mainly expressed in the antennae of honeybees and shows a strong binding ability to aldehydes in melon floral volatiles, terpenes, and benzenes in tomato floral volatiles. This may be related to the collection preference of honeybees or the location of nectar and pollen sources. The expression of AmelOBP2 in honeybee antennae was higher in the melon greenhouse than in the tomato greenhouse, and the binding ability of AmelOBP2 to melon flower volatiles was stronger than its binding ability to tomato flower volatiles. Additionally, we found that AmelOBP2 plays an important role in the recognition of e-2-hexenal, 1-nonanal, and e-2-octenal, providing a valuable reference for future research on behavioral regulation using plant volatiles. The results of this study elucidate in part the molecular mechanisms of honeybee responses to tomato and melon floral volatiles.

## Figures and Tables

**Table 1:** Binding ability of AmelOBP2 to floral volatiles of melon and tomato.

Category	Ligand Names	IC50 (μM)	K_i_ (μM)
Melon floral volatiles	dodecanal	2.35	1.05
	e-2-decenal	5.41	2.42
	e-2-hexenal	6.33	2.83
	1,3-bis (1,1-dimethyl ethyl)-benzene	6.37	2.85
	decanal	8.32	3.72
	benzaldehyde	8.37	3.74
	trans-β-ionone	9.43	4.21
	3-nonen-2-one	9.63	4.30
	benzeneacetaldehyde	10.19	4.55
	2,2,4-trimethyl-1,3-pentanediol diisobutyrate	11.01	4.92
	1-nonanal	11.77	5.26
	hexadecenoic acid	11.85	5.29
	dimethyl phthalate	12.40	5.54
	3-phenyl-2-propenal	12.74	5.69
	2,5-dimethyl-benzaldehyde	15.94	7.12
	benzenepropanal	16.34	7.30
	e-2-octenal	16.68	7.45
	1,2-dimethoxy-4-(1-propenyl)-benzene	17.20	7.68
	hexadecanoic acid, ethyl ester	28.77	12.85
	tetracosane	29.60	13.22
	nonadecane	32.96	14.72
	phytol	36.30	16.22
	heptadecane	37.33	16.68
	heneicosane	48.57	21.70
	hexadecane	68.59	30.64
Tomato floral volatiles	alpha-myrcene	6.18	2.76
	p-cymene	8.02	3.58
	β-ocimene	8.40	3.75
	gamma-terpinene	9.31	4.16
	β-caryophyllene	10.83	4.84
	1-nonanal	11.77	5.26
	2-methyl-5-(1-methyl ethenyl)-cyclohexanone	13.50	6.03
	1-methyl-4-(1-methyl ethenyl)-benzene	13.95	6.23
	3,7-dimethyl-1,6-octadien-3-ol	15.70	7.01
	2,6,6-trimethyl-2,4-cyclopentadiene-1-one	17.02	7.60
	tetradecane	18.05	8.06
	methyl benzene	18.57	8.30
	1,3-dimethyl-benzene	24.64	11.01
	3-methyl-6-(1-methyl ethyl)-2-cyclohexen-1-one	25.03	11.18

Note: IC50, the concentration of the odor standard when the fluorescence intensity of the AmelOBP2/1-NPN complex is reduced to half; Ki, binding constant.

**Table 2:** Electroantennogram response to compounds under RNAi silencing in *Apis mellifera ligustica*.

Compounds	EAG Relative Value (mV)			Silencing Efficiency (%)
	CK	NC	RNAi	
e-2-hexenal	4.91 ± 0.09 ^a^	4.81 ± 0.11 ^a^	3.21 ± 0.01 ^b^	34.64%
1-nonanal	2.96 ± 0.05 ^a^	2.86 ± 0.08 ^a^	1.85 ± 0.17 ^b^	37.28%
e-2-octenal	3.40 ± 0.01 ^a^	3.22 ± 0.10 ^a^	1.52 ± 0.05 ^b^	55.20%
2-methyl-5-(1-methyl ethenyl)-cyclohexanone	1.11 ± 0.17	1.14 ± 0.03	0.75 ± 0.13	32.48%
methyl benzene	0.74 ± 0.06	0.79 ± 0.08	0.56 ± 0.04	28.76%

Note: Different letters indicate significant differences in the same row (*p* < 0.05). Results are presented as mean ± standard error. EAG, electroantennogram; CK, sucrose solution; NC, siNC.

## Data Availability

All data generated or analyzed during this study are included in this published article.

## Appendix A

**Table A1:** Quantitative real-time polymerase chain reaction system and reaction program.

Reagent	System	Reaction Program
SYBR^®^Premix Ex Taq^TM^Ⅱ	7.5 μL	Pre-denaturation: 95 °C 10 min; 40 cycles: 95 °C 60 s, 60 °C 20 s, 72 °C 30 s. Determination of solution curve: 95 °C 10 s, 40 °C 1 min, 95 °C 1 min.
Forward Primer, 10 μM	0.6 μL	
Reverse Primer, 10 μM	0.6 μL	
cDNA	0.1 μg	
RNase Free dH_2_o	Up to15.0 μL	

**Table A2:** Primers used in this study.

Gene Name	Primer Sequences (5’-3’)
*Amel*OBP2 (qRT-PCR)	F: AACACCCTCGTCACCGTTAC
	R: CGTTTCATCACGCAGGCTTT
*β-actin*	F: ATGCCAACACTGTCCTTTCTGG
	R: GACCCACCAATCCATACGGA
*Amel*OBP2 (recombinant expression)	F: CGCCATATGATGAACACCCTCGTCACCGT
	R: CCCCTCGAGTTACGAGAACAGTTTCTCGATGT
siAmelOBP2-1	F: GCAAAGUACAUGGAGUAUUTT
	R: AAUACUCCAUGUACUUUGCTT
siAmelOBP2-2	F: GGAUCGCGAAUGAGUGCAUTT
	R: AUGCACUCAUUCGCGAUCCTT
siNC	F: UUCUCCGAACGUGUCACGUTT
	R: ACGUGACACGUUCGGAGAATT

**Table A3:** PCR reaction system and reaction program.

Reagent	Dosage	Reaction Program
Easy Taq^®^ DNA Polymerase	1.0 μL	① 95 °C, 4 min, ② 35 cycles: 94 °C, 30 s; 58 °C, 30 s; 72 °C, 30 s;
Forward Primer, 10 μM	1.0 μL	
Reverse Primer, 10 μM	1.0 μL	
cDNA	1.0 μg	③ 72 °C, 8 min; ④ 4 °C, ∞.
10×Easy Taq^®^ Buffer	5.0 μL	
2.5 mM dNTPs	4.0 μL	
ddH_2_o	Up to 20.0 μL	

**Table A4:** The digestion and ligation reaction system and reaction program.

	**Reagent**	**Dosage**
Double digestion reaction 37 °C, 1 h	PCR-recovered products and pET-30a vector	1.0 μg
	10 × K Buffer	2.0 μL
	0.1% BSA	2.0 μL
	*Nde* I	1.0 μL
	*Xho* I	1.0 μL
	ddH_2_O	Up to 20.0 μL
Ligation reaction4 °C, 24 h	pET30a	0.1 μg
	Target fragment digestion product	0.1 μg
	T4 DNA ligase	2.0 μL
	2 × Rapid Ligation Buffer	10.0 μL
	ddH_2_O	Up to 20.0 μL

**Table A5:** Standard compounds tested.

Compounds	CAS Number	Purity	Manufacturer
2-methyl-5-(1-methyl ethenyl)-cyclohexanone	7764-50-3	98%	Aladdin
p-cymene	99-87-6	≥99.5%	Aladdin
1-methyl-4-(1-methyl ethenyl)-benzene	1195-32-0	>95%	Aladdin
2,6,6-trimethyl-2,4-cycloheptadien-1-one	503-93-5	≥96%	Macklin
β-caryophyllene	87-44-5	99%	Macklin
gamma-terpinene	99-85-4	>95%	Aladdin
3,7-dimethyl-1,6-octadien-3-ol	78-70-6	98%	Aladdin
3-methyl-6-(1-methyl ethyl)-2-cyclohexen-1-one	89-81-6	>94%	TCI
methyl benzene	108-88-3	99.8%	Sigma
1,3-dimethyl-benzene	108-38-3	>99%	Aladdin
β-ocimene	13877-91-3	>90%	Sigma
tetradecane	629-59-4	>99%	Aladdin
alpha-myrcene	123-35-3	≥90%	Aladdin
1-nonanal	124-19-6	96%	Aladdin
2,5-dimethyl-benzaldehyde	5779-94-2	98%	Macklin
trans-β-ionone	79-77-6	>95%	TCI
heneicosane	629-94-7	>99%	Aladdin
3-phenyl-2-propenal	104-55-2	98%	Macklin
hexadecane	544-76-3	99%	Macklin
heptadecane	629-78-7	≥99%	Macklin
methyl palmitate	112-39-0	99%	Aladdin
2,2,4-trimethyl-1,3-pentanediol diisobutyrate	6846-50-0	98.5%	Aladdin
3-nonen-2-one	14309-57-0	≥96%	Macklin
tetracosane	646-31-1	AR	Aladdin
e-2-decenal.	3913-81-3	95%	Aladdin
e-2-octenal	2548-87-0	95%	Aladdin
decanal	112-31-2	97%	Aladdin
dodecanal	112-54-9	95%	Aladdin
nonadecane	629-92-5	98%	Macklin
1,2-dimethoxy-4-(1-propenyl)-benzene	93-16-3	>98%	Aladdin
phytol	150-86-7	>90%	Aladdin
ethyl palmitate	628-97-7	≥99%	Aladdin
1,3-bis (1,1-dimethyl ethyl)-benzene	1014-60-4	>98%	Aladdin
benzenepropanal	104-53-0	95%	Aladdin
benzaldehyde	100-52-7	≥99.5%	Aladdin
benzeneacetaldehyde	122-78-1	95%	Aladdin
e-2-hexenal	6728-26-3	98%	Aladdin
